# Stress Evaluation of Welded Joints with Metal Magnetic Memory Testing Based on Tension–Compression Fatigue Test

**DOI:** 10.3390/ma15093103

**Published:** 2022-04-25

**Authors:** Huipeng Wang, Zhiwei Xu, Dongwei Cai, Lihong Dong, Guozheng Ma, Haidou Wang, Bin Liu

**Affiliations:** 1 School of Mechanical and Electrical Engineering, Jiangxi University of Science and Technology, Ganzhou 341000, China; 6720210731@mail.jxust.edu.cn (Z.X.); 6720210730@mail.jxust.edu.cn (D.C.); 2 National United Engineering Laboratory for Advanced Bearing Tribology, Henan University of Science and Technology, Luoyang 471023, China; 3 National Key Laboratory for Remanufacturing, Army Academy of Armored Forces, Beijing 100072, China; magz0929@163.com (G.M.); wanghaidou@tsinghua.org.cn (H.W.); 4 National Engineering Research Center for Remanufacturing, Army Academy of Armored Forces, Beijing 100072, China; 5 School of Material Science and Engineering, Jiangsu University of Science and Technology, Zhenjiang 212003, China; liubindely@163.com

**Keywords:** welded joint, tension-compression fatigue test, metal magnetic memory testing, characteristic extraction, stress evaluation

## Abstract

Metal magnetic memory testing (MMMT) is an effective nondestructive technique for fatigue damage monitoring of weldments because of its capacity for stress evaluation. An experimental investigation of the effect of the applied fatigue stress on MMMT signals, including the tangential component *B_x_* and the normal component *B_z_*, during tension–compression fatigue tests in welded joints was carried out systematically. The *B_x_* and *B_z_* signals at different fatigue cycles and fatigue stresses were collected and analyzed, and the results showed that there was a peak of *B_x_* and abnormal peaks of *B_z_* that existed at the welded joint before loading. After loading, the peak of *B_x_* and the abnormal peaks of *B_z_* reversed, and the *B_x_* signals moved upward and the *B_z_* signals rotated anticlockwise dramatically in the first few fatigue cycles. After the fatigue cycle number was larger than 1000, *B_x_* and *B_z_* were stable, with very little fluctuation. In addition, the characteristics of *B_x_* signals, the mean value, and the peak value of the average of *B_x_* had an extremely significant linear relationship with the applied fatigue stress during the stable stage of the fatigue test, which indicates that MMMT is a feasible method for fatigue stress evaluation and even residual fatigue life estimation for weldments in service.

## 1. Introduction

Metal magnetic memory testing (MMMT) can not only detect already-developed defects but can also evaluate stress concentration zones (SCZs), which can evolve into macroscopic defects under the effect of an external load on the basis of the magneto-mechanical effect [1,2]. As a result of the interaction between external stress and the geomagnetic field, magnetic domains in the SCZs are reoriented, a spontaneous magnetic field near the surface can form even when the load is released, and the characteristics of the spontaneous magnetic field can be used to identify the SCZs. Because of its prominent advantage in early damage diagnosis, along with passive magnetic field measurement, simple operation, high sensitivity, time-saving, etc., MMMT has attracted extensive attention since it was proposed by Doubov in the late 1990s [3,4], and many experimental studies focusing on the relationship between the MMMT signals of ferromagnetic materials and the stress and damage status have been carried out. Static tensile tests show that characteristics of MMMT signals can indicate plastic deformation [5,6], the stress concentration degree [7,8], and the length of backside cracks [9]. Furthermore, tension–tension fatigue tests [10,11], three-point bending fatigue tests [12,13], and four-point bending fatigue tests [14] show that the characteristics of MMMT signals increase linearly with increases in crack length. Moreover, many theories have also been presented to explore the foundation of MMMT. Magnetic dipole models [15,16,17], magneto-elastoplastic coupling models [18,19], the thermo-magneto-elastoplastic coupling model [20], density functional theory [21], and the modified Jiles–Atherton model [22] have been introduced to reveal the quantitative relationship between the MMMT signals and the stress and damage of ferromagnetic materials.

Welding is commonly used as an important mechanical structure connection method for equipment manufacturing in the aerospace, pressure vessel, and petroleum pipeline industries, among others [23,24]. Additionally, for safety assurance, welded structures are usually inspected by conventional nondestructive testing methods (visual testing, radiography testing, ultrasonic testing, magnetic particle testing, magnetic flux leakage testing, etc.) to detect the already-developed defects resulting from the welding process [25]. However, stress concentration introduced during the welding process, which cannot be detected by conventional NDT methods, plays a much more dangerous role in the service life of welded structures. During service, fatigue cracks usually initiate from the SCZs of weldment under the effect of an applied alternative load, ultimately causing the failure of the weld structure. Therefore, it is vital to inspect and monitor the fatigue stress of weldments.

MMMT has drawn much interest in the inspection and evaluation of weldment for its great advantages in SCZ detection. Additionally, it has been widely applicated in the engineering field, especially in weldment detection and evaluation [26,27,28], and qualitative and quantitative research has also been carried out by many researchers. Tensile and bending tests of butt welds show that MMMT can predict weld failure with early warnings [29], and three-point bending fatigue tests of weld joints show that the maximum gradient of the normal component of MMMT signals increases exponentially with an increase in crack length [30]. Experimental investigations also show that MMMT can detect buried welding cracks effectively [31].

However, it should be noted that most MMMT research and applications in weldment evaluation have focused on macroscopic defect measurement or crack propagation detection, and far fewer works have been concerned with early damage or stress evaluation. For these interests, the work presented here focused on the magnetic behavior of welded joints during fatigue tests, analyzing variations in both the tangential and the normal component of MMMT signals during the fatigue tests; the relationship between the characteristics of MMMT signals and fatigue stress is discussed, and an MMMT stress evaluation method for welded joints is proposed.

## 2. Experimental Procedure

### 2.1. Specimen Preparation

Butt welded joints with a base metal of Optim 900QC steel, welding wire of wire CARBOFIL NiMoCr, and shielding gas of CO_2_ were investigated in this study. Optim 900QC steel, a Swiss high-strength structural steel with excellent weldability and bendability, is a low-alloy quenched and tempered steel that can be welded directly, and no additional tempering treatment is required after welding. Wire CARBOFIL NiMoCr is a kind of copper-plated solid wire that is suitable for gas-shielded welding of high-strength steel. The chemical compositions and mechanical properties of the base metal and welding wire are shown in Table 1 and Table 2, respectively.

**Figure 1:**
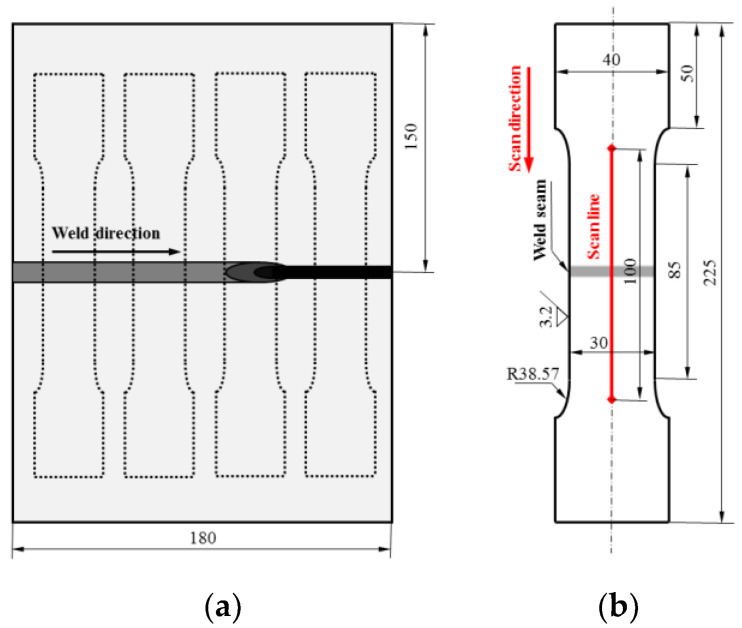
Sketch of specimen preparation. (**a**) Welding direction and sampling location, and (**b**) geometry and dimensions of the fatigue specimen. All dimensions are in mm.

Sheets of 900QC steel with a nominal thickness of 5 mm were investigated, and they were machined into a size of 150 mm × 180 mm parallel to the welding direction. The welded joints were fabricated by the CO_2_ gas-shielded arc welding technique automatically, and the welding parameters are shown in Table 3. After welding, the fatigue specimens of the welded joints of 900QC steel were machined according to the Chinese industrial standard NB/T 47014-2011 welding procedure qualification for pressure equipment, and the sampling location and specimen configuration are shown in Figure 1.

Non-destructive testing methods, both visual testing and radiographic testing, were performed to examine the quality of all the specimens, and only specimens with no examined defects were selected for the fatigue tests. The selected specimens were induction-demagnetized by a handheld induction demagnetizer, WLM-TB60, with an effective demagnetization rate of 1 μT to reduce the magnetic field interference induced by joint fabricating and specimen machining before the tests.

### 2.2. Experimental Instruments

Tension–compression fatigue tests of welded joints were performed at room temperature on a servo-hydraulic MTS 809 testing system, whose dynamic load error is ±1% and fatigue loading frequency is 0~50 Hz.

The MMM signals, both the tangential component *B_x_* and the normal component *B_z_*, of the specimens were collected by a 3D gaussmeter, the CH-3600 Gaussmeter, with a resolution of 0.1 μT. *B_x_* is parallel to the surface of the specimen and along the scanning direction of the probe, while *B_z_* is perpendicular to the surface of the specimen. A non-magnetic three-dimensional electrical control displacement instrument was employed to fix the probe firmly and move it automatically with a constant speed and lift-off value to avoid interference from external magnetic fields and manual operation errors.

### 2.3. Experimental Arrangement

The specimen was first placed on the electrical control displacement instrument along the south–north direction, and the initial MMMT signals of the scan line were measured with a probe scanning speed of 10 mm/s and a lift-off value of 1 mm. After that, the specimen was clamped between the gripers vertically, and the fatigue test was performed. When loaded to a preset fatigue cycle number, the specimen was removed from the gripers and placed on the displacement instrument, and the MMMT signals were collected in the same way as the initial MMMT signals were collected. Then, the specimen was loaded to another predetermined fatigue cycle number, and the above procedure was repeated until the specimen failed.

## 3. Results and Discussion

Fatigue tests of specimens with maximum fatigue stress of 150, 200, 300, and 400 MPa, respectively, were carried out according to the mechanical strength of welded joints, and the corresponding fatigue cycle numbers of each specimen were 194,014, 80,792, 19,262, and 10,026 in sequence. The stress ratio and frequency of all fatigue tests were the same, −1 and 10 Hz, respectively, to facilitate comparisons. For convenience, the specimens were named specimens 1, 2, 3, and 4.

### 3.1. MMMT Signals Variation during Fatigue Test

The variation law of MMT signals of specimens with different maximum fatigue stress showed the same tendency. Therefore, only the results of specimen 3 are presented in this section.

**Figure 2:**
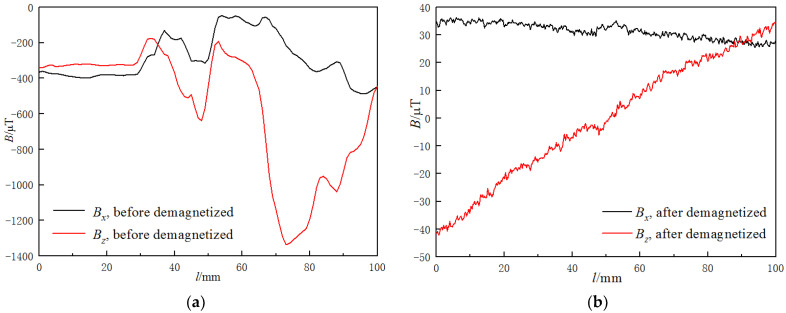
MMMT signals of the specimen (**a**) before and (**b**) after demagnetization.

Figure 2 shows the MMMT signals before and after induction–demagnetization. It can be seen in Figure 2a that the MMMT signals were highly fluctuant and varied from −1400 μT to 0 μT before being demagnetized. There were abnormal peaks around the welded joint, but one cannot judge the existence of a welded joint because of the great fluctuation of the magnetic field.

The MMMT signals became much weaker and more regular after induction–demagnetization, as shown in Figure 2b. The *B_x_* signal is almost horizontal with an upward peak at the center of the scan line, which represents the right part of the welded joint. The mean value of *B_x_* was 31.47 μT, and the peak value was 35.1 μT. The *B_z_* signal is an inclined line ranging from −40.0 to 32.0 μT with a slope value of −0.7065 μT/mm, and there are two weak abnormal peaks around the welded joint, with a negative peak on the left and a positive one on the right.

The strong magnetic field relates to the welding, machining, and transporting process. During welding and machining, the specimens were fixed by an electromagnet, which introduced an additional magnetic field to the specimen. In addition, the specimen could also be magnetized by other magnetic materials during transportation.

The magnetic field interference could be reduced significantly by induction demagnetization. After demagnetization, magnetic moments became aligned with the direction of the geomagnetic field, and magnetic flux leakage took place around the welded joint as a result of magnetic permeability differences between the base metal and the welding wire. Hence, there was an upward peak of the *B_x_* signal, while there were two weak abnormal peaks, with a negative one on the left and a positive one on the right of the *B_z_* signal around the welded joint.

**Figure 3:**
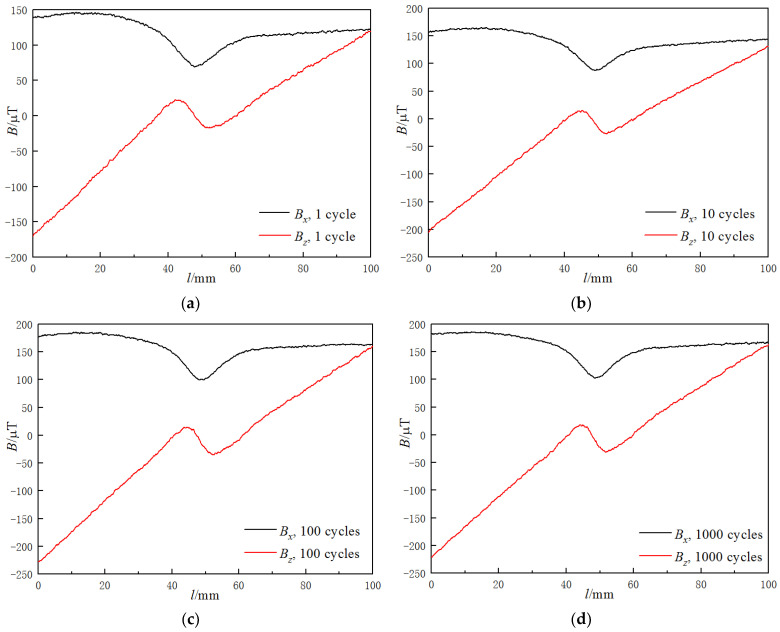

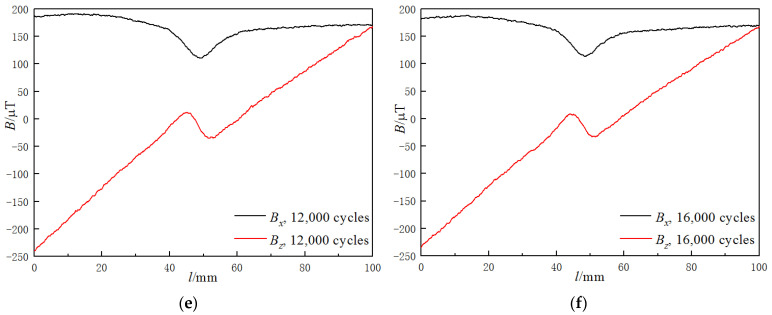
MMMT signals of welded joint under different fatigue cycle numbers: (**a**) 1 cycle; (**b**) 10 cycles; (**c**) 100 cycles; (**d**) 1000 cycles; (**e**) 8000 cycles; (**f**) 16,000 cycles.

After that, a tension–compression fatigue test was carried out, and the MMM signals of the specimen were collected during the test. The specimen fractured at 19,262 cycles, and the MMM signals of the specimen during the fatigue test are shown in Figure 3.

The signal curves changed obviously after loading, as shown in Figure 3a. The *B_x_* signal elevated upward, and there was a downward peak with a peak value of 69.2 μT at the welded joint. Both halves of the *B_x_* signal were horizontal, but the amplitudes were not the same, and the mean value of the left half was 137.62 μT, while that of the right half was 116.61 μT. The *B_z_* signal rotated anticlockwise with a positive abnormal peak on the left of the welded joint and a negative one on the right. The *B_z_* signal ranged from −170.3 to 119.8 μT, and both halves were inclined, but they were not on the same inclined line, with a slope value of 4.641 μT/mm on the left and 2.900 μT/mm on the right, and the slope of the *B_z_* signal between the abnormal peaks was −0.707 μT/mm.

The *B_x_* signal continued elevating upward, and the *B_z_* signal continued rotating anticlockwise as the fatigue cycle number increased for the first few fatigue cycles, as shown in Figure 3b,c. After that, when the fatigue cycle number became greater than 100, the MMMT signals were stable, with a little fluctuation as the fatigue cycle number increased, as shown in Figure 3d–f.

When loaded, the specimen was magnetized by the stress-induced magnetic field, and the amplitude of the MMMT signals increased. When the applied stress is greater than 70 MPa, the polarity of the stress-induced magnetic field is opposite to the geomagnetic field [32]. As a result, the MMMT signal around the welded joint reversed even one fatigue cycle was executed, with a downward peak of *B_x_*, a positive abnormal peak on the left, and a negative one on the right of *B_z_*.

Under the influence of the fatigue stress-induced magnetic field, magnetic domains tend to align themselves in the direction of the stress-induced magnetic field; the 180° domains, whose directions of moment are parallel to the direction of the magnetic field, grow, while the 90° domains, whose moment directions are perpendicular to the magnetic field, are reduced [33]. As a result, the magnetization of the specimen changes, and the magnetic flux leakage increases. Hence, the *B_x_* signal elevated upward significantly, and the *B_z_* signal rotated anticlockwise dramatically during the first few fatigue cycles, as shown in Figure 3b,c.

The fatigue stress-induced magnetic field reached the saturation limit, and no more irreversible magnetic domain movement occurred after 100 fatigue cycles. While there was still a reversible domain change, domain wall rotation [34] occurred during the following fatigue test. Hence, when the fatigue test proceeded, the MMMT signals were stable with a little fluctuation, as shown in Figure 3d–f.

**Figure 4:**
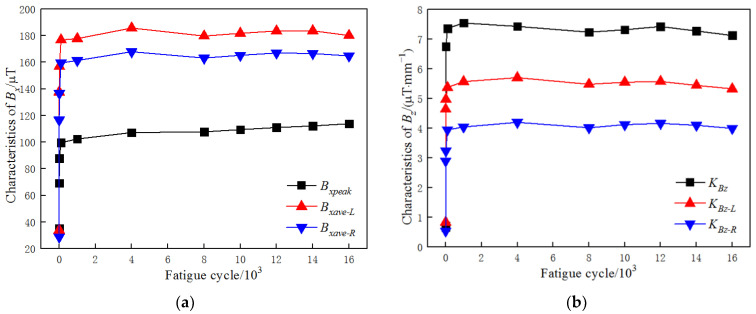
Characteristics variation of (**a**) *B_x_* signals and (**b**) *B_z_* signals during the fatigue test.

It can be seen in Figure 3 that the distribution of the MMMT signals was regular during the fatigue test. There was a peak at the welded joint of the measured line for the *B_x_* signal, and both halves beyond the welded joint were horizontal, while the amplitudes were different. There were two abnormal peaks around the welded joint for the *B_z_* signal, and both halves were inclined, while the slopes were not the same. Thus, it was feasible to extract the characteristics of the MMMT signals for further investigation. According to the features of the MMMT signals, the characteristics were defined as follows: $B_{xpeak}$ is the peak value of the *B_x_* signal; $B_{x_{ave}-L}$ and $B_{x_{ave}-R}$ are the mean values of the left and right side of the *B_x_* signal, respectively; $K_{G}$ is the gradient between the abnormal peaks of the *B_z_* signal, $K_{B_{z}-L}$ and $K_{B_{z}-R}$ are the slopes of the left and right sides of the *B_z_* signal, respectively. The characteristic variations of the MMMT signals during the fatigue test are shown in Figure 4.

It can be seen in Figure 4 that all characteristics of the *B_x_* and *B_z_* signals show the same variation tendency. In the first few fatigue cycles, they increased dramatically, and when the fatigue cycle number was more than 100, they were stable with a little fluctuation. It should also be noted that $B_{x_{ave}-L}$ and $B_{x_{ave}-R}$ had almost the same variation except for the value during the fatigue test, and the same was true for $K_{B_{z}-L}$ and $K_{B_{z}-R}$.

### 3.2. Characteristics of MMMT Signals under Different Fatigue Stress

**Figure 5:**
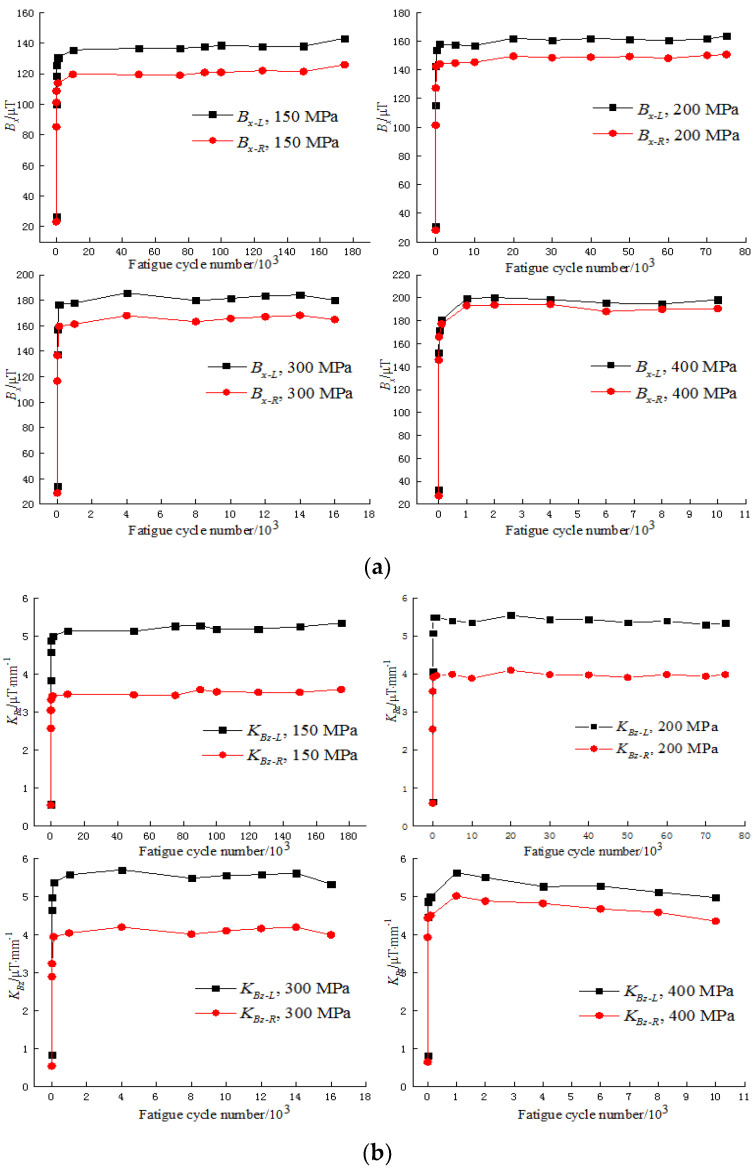
Characteristic variation of (**a**) *B_x_* signals and (**b**) *B_z_* signals of different specimens during the fatigue test.

The MMMT signals of specimens 1, 2, and 4 during the fatigue tests were also collected, and the variation laws were similar to those of specimen 3. Therefore, the same characteristics, including $B_{xpeak}$, $B_{x_{ave}-L}$, $B_{x_{ave}-R}$, $K_{G}$, $K_{B_{z}-L}$, and $K_{B_{z}-R}$, were extracted. As shown in Section 3.1, $B_{x_{ave}-L}$ and $B_{x_{ave}-R}$ and $K_{B_{z}-L}$ and $K_{B_{z}-R}$ of specimen 3 showed the same variation during the fatigue test except for the value, so it was important to find out whether the situations were the same for other specimens; the variations of the mentioned characteristics of all specimens are shown in Figure 5.

It can be seen from Figure 5 that $B_{x_{ave}-L}$ and $B_{x_{ave}-R}$ and $K_{B_{z}-L}$ and $K_{B_{z}-R}$ shared the same variation law for each specimen. For quantitative description, the cross-correlation coefficient ρ was introduced to evaluate the relationship between $B_{x_{ave}-L}$ and $B_{x_{ave}-R}$ and between $K_{B_{z}-L}$ and $K_{B_{z}-R}$. ρ is defined according to statistics:

(1) $$\rho_{x,y}=\frac{\sum_{1}^{n}\left(X_{i}-\bar{X}\right)\left(Y_{i}-\bar{Y}\right)}{\sqrt{\sum_{1}^{n}{\left(X_{i}-\bar{X}\right)}^{2}\sum_{1}^{n}{\left(Y_{i}-\bar{Y}\right)}^{2}}},$$

where $\bar{X}$ and $\bar{Y}$ are the arithmetic mean values of X and Y, respectively.

As only the variation of the MMMT signals during the stable stage was concerned, data collected when the fatigue cycle number was less than 100 were ignored, and the cross-correlation coefficients of $B_{x_{ave}-L}$ and $B_{x_{ave}-R}$ and $K_{B_{z}-L}$ and $K_{B_{z}-R}$ of each specimen are shown in Table 4.

It can be seen in Table 4 that, for each specimen, $B_{x_{ave}-L}$ and $B_{x_{ave}-R}$ and $K_{B_{z}-L}$ and $K_{B_{z}-R}$ were highly correlated with each other during the fatigue tests. Therefore, the new characteristics $B_{x_{ave}}$ and $K_{B_{z}}$ were introduced for simplification. $B_{x_{ave}}$ is the mean value of $B_{x_{ave}-L}$ and $B_{x_{ave}-R}$, while $K_{B_{z}}$ is the slope of the angular bisector of line $y=K_{B_{z}-L}\cdot x$ and $y=K_{B_{z}-R}\cdot x$, as shown in Equation (2).

(2) $$B_{x_{ave}}=\frac{B_{x_{ave-L}}+B_{x_{ave-R}}}{2}\;K_{B_{z}}=\frac{K_{B_{z}-L}\cdot K_{B_{z}-R}-1+\sqrt{{\left(1-K_{B_{z}-L}\cdot K_{B_{z}-R}\right)}^{2}+{\left(K_{B_{z}-L}+K_{B_{z}-R}\right)}^{2}}}{K_{B_{z}-L}+K_{B_{z}-R}},$$

**Figure 6:**
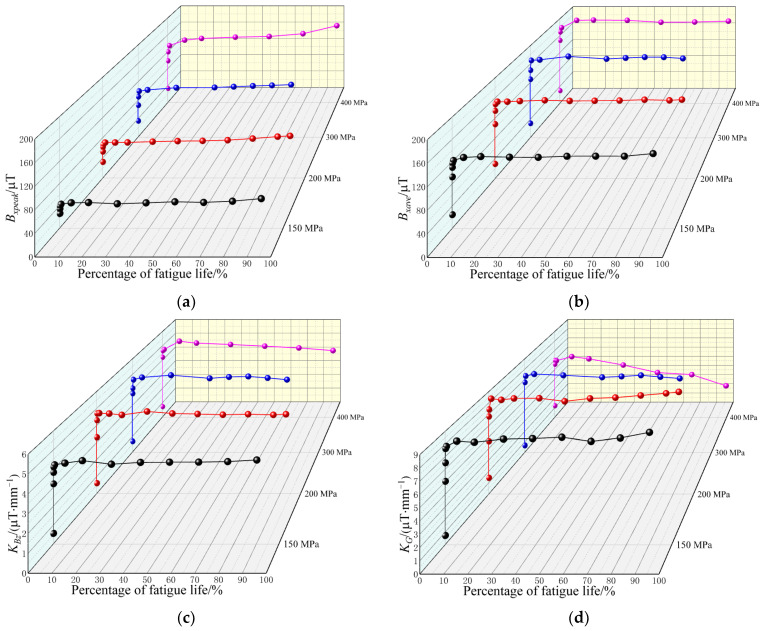
Characteristic variations of MMMT signals during the fatigue tests (**a**) $B_{xpeak}$; (**b**) $B_{xave}$; (**c**) $K_{B_{z}}$; (**d**) $K_{G}$.

Therefore, the number of characteristics can be reduced to four, i.e., $B_{xpeak}$, $B_{xave}$, $K_{B_{z}}$ and $K_{G}$, and their variations during the fatigue tests are shown in Figure 6.

Figure 6a shows the variation in $B_{xpeak}$ for all the specimens during the fatigue tests. $B_{xpeak}$ of the specimens increased significantly with the fatigue cycle number at first, and then became stable and fluctuated a little as the fatigue test proceeded. It also can be seen that when the specimens were about to fracture, the amplitudes of $B_{xpeak}$ of the last measurement of specimen 1 (175,000 cycles, 90.20% of fatigue life), specimen 2 (75,000 cycles, 92.83% of fatigue life), and specimen 4 (10,000 cycles, 99.74% of fatigue life) were much larger than those measured before, while that of specimen 3 (16,000 cycles, 83.07% of fatigue life) was not.

It can be seen in Figure 6b that $B_{xave}$ of all the specimens during the fatigue tests showed almost the same variation: they increased dramatically in the first few fatigue cycle numbers, and after that, they were stable with a little fluctuation.

It can be concluded from Figure 6a,b that when the specimen is about to fracture (above 90% of fatigue life), $B_{xave}$ barely changes, while $B_{xpeak}$ changes obviously. This phenomenon could be a significant criterion for failure pre-warning of welded joints.

As shown in Figure 6c, $K_{B_{z}}$ of all the specimens increased as the fatigue cycle number increased in the first few fatigue cycle numbers, and then became stable with a little fluctuation until the specimens failed. By contrast, the variations in $K_{G}$ showed the same tendency, as shown in Figure 6d, except that of specimen 4. $K_{G}$ of specimen 4 increased with the fatigue cycle at the beginning of the fatigue test and then fluctuated much more obviously until the specimen fractured.

**Figure 7:**
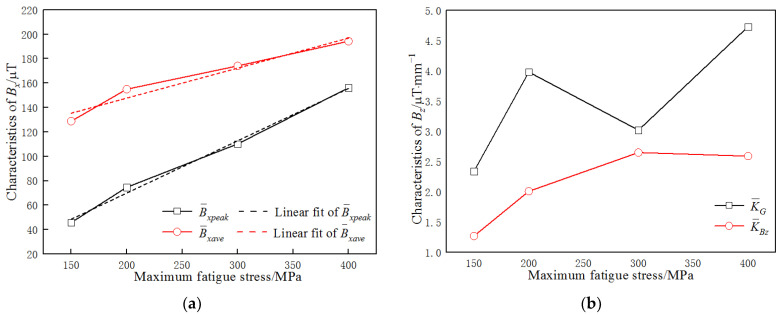
Characteristic variation of MMMT signals with the maximum fatigue stress (**a**) *B_x_* signals, (**b**) *B_z_* signals.

To explore the relationship between the characteristics of MMMT signals and the fatigue stress, the mean values of the characteristics of the MMMT signals, ${\bar{B}}_{xave}$, ${\bar{B}}_{xpeak}$, ${\bar{K}}_{B_{z}}$, and ${\bar{K}}_{G}$, during the stable stage of the fatigue tests (when fatigue cycle number was larger than 100) were analyzed, and their variations with maximum fatigue stress are shown in Figure 7. The values of ${\bar{B}}_{xpeak}$ of the last measurements of specimens 1, 2, and 4 were neglected, as they were much larger than those measured before (in other words, they were about to fracture).

It can be seen in Figure 7 that there is a linear relationship between ${\bar{B}}_{xave}$ and the applied maximum fatigue stress σ and between ${\bar{B}}_{xpeak}$ and σ. The functions can be expressed as follows:

(3) $${\bar{B}}_{xpeak}=0.4282\cdot \sigma -15.71\;{\bar{B}}_{xave}=0.2458\cdot \sigma +98.52,$$

Unfortunately, there is no obvious relationship between ${\bar{K}}_{B_{z}}$ or ${\bar{K}}_{G}$ and σ.

It can be seen from Equation (3) that there was a linear relationship between ${\bar{B}}_{xave}$ or ${\bar{B}}_{xpeak}$ and the applied maximum fatigue stress σ of the welded joints in this work. Therefore, it is feasible to evaluate σ by the measurement of the *B_x_* signal, as shown in Equation (4):

(4) $$\sigma =37.927+2.323\cdot {\bar{B}}_{xpeak}\;\sigma =-370.397+3.882\cdot {\bar{B}}_{xave},$$

It should be noted that the premise of the σ calculation by Equation (4) is that the fatigue stress ratio is −1. As a matter of fact, fatigue with a stress ratio that is not −1 is commonly experienced in both experimental research and engineering application. In this case, the Goodman formula can be introduced for equivalent transformation. The Goodman formula is shown in Equation (5):

(5) $$\sigma_{a}=\sigma_{-1}\left(1-\frac{\sigma_{m}}{\sigma_{b}}\right),$$

where $\sigma_{a}=\left(\sigma_{max}-\sigma_{min}\right)/2$, is the stress amplitude, $\sigma_{m}=\left(\sigma_{max}+\sigma_{min}\right)/2$, is the mean stress, and $\sigma_{b}$ is the tensile strength of the material.

When measurement of *B_x_* is executed, the characteristics ${\bar{B}}_{xave}$ and ${\bar{B}}_{xpeak}$ can be extracted. Then, the applied maximum fatigue stress $\sigma_{-1}$ when the stress ratio is −1 can be calculated. The actual stress amplitude $\sigma_{a}$ can be calculated according to Equation (5).

## 4. Conclusions

The systematic investigation of MMMT signal variation of welded joints under different fatigue cycle numbers during the whole tension–compression fatigue tests was presented in this work. There was a peak at the welded joint for the *B_x_* signal, and both halves beyond the welded joint were horizontal, while the amplitudes were different. There were two abnormal peaks around the welded joint for the *B_z_* signal, and both halves were inclined, while the slopes were not the same. The following conclusions can be drawn:

(1) The *B_x_* signal elevated upward, and the *B_z_* signal rotated counterclockwise dramatically with an increase in the fatigue cycle number in the first few fatigue cycles; after that, both signals were stable with a little fluctuation.
(2) When the welded joint is about to fracture (about 90% of the fatigue life in this work), the mean value of the *B_x_* signal barely changes, while the peak value increases obviously, which can be used as a service status pre-warning for weld structures.
(3) Fatigue stress of welded joints can be estimated and monitored by the mean value and peak value, i.e., ${\bar{B}}_{xave}$ and ${\bar{B}}_{xpeak}$, of the *B_x_* signal.

## Figures and Tables

**Table 1:** Chemical compositions of the base metal and welding wire (in wt.%).

Brand	C	Si	Mn	P	S	Cr	Ni	Mo	Cu	Ti
Optim 900QC steel	0.091	0.27	1.8	0.009	0.002	1.11	0.08	0.152	-	-
CARBOFIL NiMoCr	0.096	0.41	1.34	0.0078	0.014	0.23	1.0	0.2	0.1	0.0021

**Table 2:** Mechanical properties of the base metal and welding wire.

Brand	Yield Strength/MPa	Ultimate Strength/MPa	Elongation/%
Optim 900QC steel	960	1107	11
CARBOFIL NiMoCr	≥690	900	≥17

**Table 3:** Welding parameters for 900QC steel.

Weld Seam	Current/A	Voltage/V	Gas Flow/L·min^−1^
Backing weld	100~110	19~20	15
Capping weld	180~200	22~24	15

**Table 4:** Cross-correlation coefficients of the characteristics of all the specimens.

	Specimen 1	Specimen 2	Specimen 3	Specimen 4
$\rho_{B_{x_{ave}-L},B_{x_{ave}-R}}$	0.9919	0.9728	0.9684	0.9757
$\rho_{K_{B_{z-L}},K_{B_{z-R}}}$	0.8846	0.8289	0.8828	0.9132

## Data Availability

Not applicable.
